# Effect of Multi-Directional Forging on Microstructure and Mechanical Properties of Dual-Phase Mg-8Li-3Al-0.3Si Alloy

**DOI:** 10.3390/ma18081829

**Published:** 2025-04-16

**Authors:** Pengcheng Tian, Cuiju Wang, Kaibo Nie, Yaniu Li, Kunkun Deng

**Affiliations:** Shanxi Key Laboratory of Magnesium Matrix Materials, College of Materials Science and Engineering, Taiyuan University of Technology, Taiyuan 030024, Chinaniekaibo@tyut.edu.cn (K.N.);

**Keywords:** Mg-Li alloy, multi-directional forging, DRX, mechanical properties, work hardening behavior, work softening behavior

## Abstract

The Mg-8Li-3Al-0.3Si dual-phase alloy (LA83-0.3Si) was subjected to six multi-directional forging (MDF) passes in the present work, then its microstructure, mechanical properties, and work hardening and work softening effects were examined and analyzed. The results indicate that the continuous dynamic recrystallization (CDRX) mechanism of the LA83-0.3Si dual-phase alloy gradually transitioned to a discontinuous dynamic recrystallization (DDRX) mechanism as the temperature increased after MDF. This temperature change induced a transition in the basal texture from bimodal to multimodal, significantly reducing the texture intensity and weakening the alloy’s anisotropy. At 310 °C, the AlLi phase nucleated into coated particles to stabilize the structure. Additionally, the increase in the forging temperature weakened the synergistic deformation capability of the α/β phases, while the hardening behavior of the β-Li phase provided a nucleation pathway for dynamic recrystallization (DRX). MDF significantly enhanced the strength and ductility of the LA83-0.3Si alloy. The alloy’s strength continued to rise, while elongation decreased as the forging temperature increased. The ultimate tensile strength (UTS) and elongation (EL) reached 267.8 MPa and 11.9%, respectively. The work hardening effect increased with the forging temperature, whereas the work softening effect continuously diminished, attributed to the enhanced hardening behavior of the β phase and the reduced ability to transfer dislocations.

## 1. Introduction

Mg-Li alloys, with their high specific strength and specific stiffness, as well as outstanding impact resistance at low temperatures, have become the ideal choice for lightweight materials in many fields, such as aviation, aerospace, automotive manufacturing, and electronics [1,2,3,4]. Depending on the Li content, Mg-Li alloys can be categorized into single-phase and two-phase structures. When the Li content is lower than 5.7 wt.%, the Mg-Li alloy consists of a single α phase, which has high strength but relatively poor plasticity. When the Li content is higher than 10.3 wt.%, the Mg-Li alloy consists of a single β phase, which significantly improves the plasticity but reduces the strength, and when the Li content is increased to 5.7–10.3 wt.%, the Mg-Li alloy consists of an α and β dual-phase, which takes into account the strength of the α phase and the plasticity of the β phase, and has better strength and toughness [5,6,7,8]. However, compared with traditional magnesium alloys, such as AZ31, the absolute strength of Mg-Li alloys is relatively low, which restricts their application range to a certain extent [4,9].

Alloying represents a critical strategy for enhancing the mechanical properties of Mg-Li alloys. Al is the preferred element for strengthening Mg-Li alloys due to its high solubility in the α-Mg matrix. It facilitates the formation of AlLi and MgLi_2_Al precipitates, contributing to second-phase strengthening [10,11]. Simultaneously, the incorporation of Al elevates the critical resolved shear stress (CRSS) of basal, prismatic, and pyramidal slip systems, while reducing the CRSS of {10$\bar{1}$2} deformation twins. This modification enhances the yield strength and hardening rate of the as-cast Mg-Li alloy [3,12,13]. However, the incorporation of Al often induces aging softening, leading to a reduction in the alloy’s strength [14,15,16].

To stabilize the structure, the addition of Si, with its high melting point of 1410 °C, has become a key area of research exploration. Shi et al. [17] introduced Si into Mg-8Li alloys using an Al-12.6Si eutectic alloy, achieving a 100% increase in tensile strength compared to the as-cast Mg-8Li alloy. Zhao et al. [18] observed that adding an Al-Si hypereutectic forms a stable phase in the dual-phase alloy. Additionally, the dispersed Si phase serves as an effective nucleation site for recrystallization, enhancing the alloy’s mechanical properties. Currently, the introduction of Si into alloys is commonly achieved through the use of Al-Si hypereutectic systems. However, due to the large size of Si atoms, which are inherited into the as-cast alloy during the fabrication process of Mg-Li alloys, the strengthening efficiency is significantly compromised. Consequently, identifying how to obtain refined and uniformly distributed Si phases that could enhance mechanical properties has become a critical research focus for further development of Mg-Li alloy systems.

Hot deformation is an effective approach to further enhance the mechanical properties of alloys [4,19,20,21,22,23,24]. Compared with a single deformation method, MDF is a method to achieve microstructure refinement before rolling or extrusion [25,26,27,28,29]. A researcher formed a bimodal microstructure consisting of dynamically recrystallized (DRXed) fine grains and non-dynamic recrystallization (unDRXed) coarse grains after the low-temperature MDF of a Mg-2.76Li-3Al-2.6Zn-0.39Y alloy, with an enhancement of the UTS by about 42% [30]. Cao et al. [19] found that after six passes with MDF of the α/β dual-phase Mg-6.4Li-3.6Zn-0.37Al-0.36Y alloy, the UTS increased from 113 MPa to 207 MPa, and the coordination effect of the β phase on the α phase made the α-Mg grain refinement uniform and the fine grain strengthening effect more significant. The significant influences exerted by MDF on the microstructure and mechanical properties of Mg-Li alloys can be clearly observed. However, limited studies have been conducted on the effects of the forging temperature, which are closely associated with the microstructural evolution and mechanical properties of α/β dual-phase Mg-Li alloys. Furthermore, the dynamic recrystallization (DRX) mechanism and work hardening–work softening behaviors that characterize these alloys remain to be systematically investigated.

In this paper, an Al-27Si hypereutectic alloy with a fine Si phase was prepared by a spray deposition method, and a Mg-8Li-3Al-0.3Si (LA83-0.3Si) alloy was prepared using vacuum resistance melting equipment. Subsequently, the as-cast alloy was subjected to six passes of MDF. The effects of the forging temperature on the microstructure, mechanical properties, and work hardening and work softening behavior of the alloy were analyzed. The DRX behavior of LA83-0.3Si alloy and the work hardening and work softening behavior under the combined action of α/β were discussed.

## 2. Materials and Methods

The raw materials for the LA83-0.3Si alloy included pure magnesium ingot (99.9 wt.%, Shanxi Yinguang Huasheng Magnesium Industry Co., Ltd., Shanxi, China), pure aluminum ingot (99.9 wt.%, Shanxi Yinguang Huasheng Magnesium Industry Co., Ltd, Shanxi, China), pure lithium particles (99.99 wt.%, Zhongnuo New Materials (Beijing) Technology Co., Ltd., Beijing, China), and an Al-27Si hypereutectic (Jiangsu Haoran Spray Forming Alloy Co., Ltd., Jiangsu, China). The Al-27Si master alloy was prepared using a spray deposition process. As shown in the Supplementary File, Figure S1 shows the BSE images and EDS results of the Al-27Si master alloy. The schematic diagram of the alloy-melting equipment (Mengting Chengdu Instruments and Equipment Co., Ltd., Sichuan, China) is shown in Figure S2a. The melting process was conducted under an argon-and-SF6 atmosphere. The alloy ingot was melted at 720 °C, stirred using a stirring paddle for 3 min, and held at the target temperature for 10 min before being poured into a preheated casting mold maintained at 250 °C to form the alloy ingot. Subsequently, a 30 mm × 30 mm × 60 mm MDF sample was sectioned from the ingot using wire electrical discharge machining. During the deformation process, the holding time for each pass was maintained at 20 min, with a single pass strain of 0.8, the pressing speed controlled at 2.5 mm/s, and the pressure of 250 kN applied for 20 s. After the completion of each pass, the sample was quenched for thermal stabilization. The MDF process is schematically illustrated in Figure S2b. The LA83-0.3Si alloy was subjected to isothermal MDF for six passes at 250 °C, 280 °C, and 310 °C, and the processed samples were designated as MDF250, MDF280, and MDF310, respectively.

The microstructures of all the materials were characterized using scanning electron microscopy (SEM, JSM-IT700HR, JEOL, Tokyo, Japan) and transmission electron microscopy (TEM, JEM-2100F, JEOL, Tokyo, Japan) equipped with Oxford energy-dispersive spectroscopy (EDS, OXFORD, Britain, UK). In the Supplementary File, Figure S2c, three orthogonal directions of the MDF-processed alloy were specified, which was bisected along blue, dashed lines, with one half being used for microstructural characterization. The observation planes for OM, BSE, and EBSD analyses were identified as the forging direction–normal direction plane (FD-ND), which is perpendicular to the transverse direction. Prior to the experiments, the BSE samples were sequentially polished using 800#, 2000#, and 4000# grit water sandpapers, followed by surface etching with 3% nitric acid ethanol solution for 7–10 s. The β-Li phase and secondary phases that were selected using Photoshop (Adobe Photoshop 2022) and their volume fractions were subsequently determined by Image-Pro Plus software (Image-Pro Plus 6.0). To ensure the accuracy of the experimental results, a minimum of three scanning images were statistically analyzed. For the EBSD analysis, the samples were prepared via electrolytic polishing using an AC2 electrolyte solution. The polishing process was conducted at a temperature of −30 °C, with a voltage of 20 V, a current of 0.02–0.03 A, and a polishing duration of 60–120 s. The EBSD analysis was conducted using AZtecCrystal software during which the texture distribution mapping magnification was set to 200×. The TEM samples were initially polished to a thickness of less than 50 μm using 4000# grit water sandpaper, followed by ion thinning. The ion thinning was conducted at a voltage of 5.0 keV, with the ion beam incident at an angle of 10° to the sample surface. Upon the appearance of fine perforations, the ion beam angle was adjusted to 5°, and thinning continued for an additional 30 min. The phase composition was determined using an X-ray diffractometer (XRD, RIGAKU-SMARTLA, RIGAKU, Tokyo, Japan) with a scanning range of 20° to 80° and a scanning speed of 5°/min. The XRD phase analysis was performed using MDI Jade 6.0 and HighScore Plus software.

The room temperature tensile test was conducted using an electronic universal tensile testing machine (MTS E45.105, MN, USA). To reflect the mechanical property consistency of the material, three positions were vertically selected from the wire-cut remaining half (as marked in Figure S2c), from which dog-bone-shaped tensile specimens that were compliant with the ASTM E8 Subsample were prepared in the FD-TD plane, where the mechanical loading alignment was ensured. The tensile speed was set at 0.5 mm/min. Stress relaxation tests were also performed on the same equipment, with an initial cycle strain of 1.2%, a strain interval of 0.8%, and an unloading duration of 10 min. The surface hardness was measured using an HVT-1000 microhardness tester (HVT-1000, Guangdong, China) with a test force of 4.9 N and a dwell time of 10 s. The alloy’s XPM test was conducted using a Hysitron TI 980 nanoindentation instrument (BURKER, Billerica, MA, USA) over a 12 × 12 square grid. The spacing between adjacent test points was 5 μm, with a test load of 8000 μN. The loading, holding, and unloading times were 2 s, 5 s, and 2 s, respectively.

## 3. Results

### 3.1. Microstructure of LA83-0.3Si Alloy

**Figure 1:**
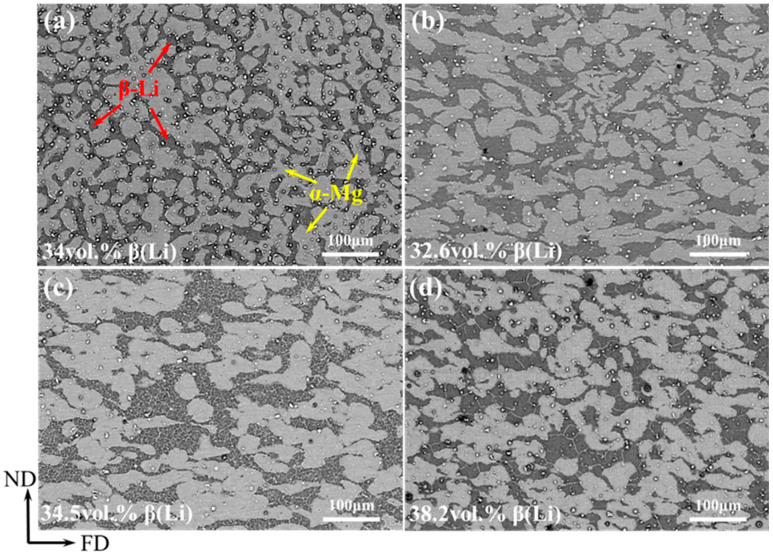
Low-magnification BSE images of LA83-0.3Si alloy before and after MDF: (**a**) as-cast, (**b**) MDF250, (**c**) MDF280, (**d**) MDF310.

The low-magnification BSE images of the LA83-0.3Si alloy before and after MDF are depicted in Figure 1. The alloy primarily consists of a gray α-Mg phase and a black β-Li phase. Figure 1a illustrates the microstructure of the as-cast alloy, where the β-Li phase exhibits an interconnected network distribution. The volume fraction of the β-Li phase is approximately 34%, as determined by Image Pro Plus software. In Figure 1b, it is evident that post-MDF, the β-Li phase is elongated along the forging direction (FD) and has coarsened, with a volume fraction of about 32.6%. As the forging temperature increases, the α-Mg phase undergoes spheroidization, the β-Li phase further enlarges, and the phase boundaries bend inward. This is due to the instability of the surface curvature of the α/β phase boundary at elevated temperatures, where accumulated interfacial energy during the MDF process drives the grain boundaries to adopt a curvature-like spherical configuration [31]. The volume fraction of β-Li measured by software is about 34.5%. When the forging temperature reaches 310 °C, the degree of the bending of the phase boundary of the β phase in the alloy increased, while more fine β phases appeared, which were counted to have a volume fraction of about 38.2%. It can be observed that with the continuous rise in the forging temperature, the volume fraction of the β phase gradually increases, leading to an increase in the α/β phase interface.

**Figure 2:**
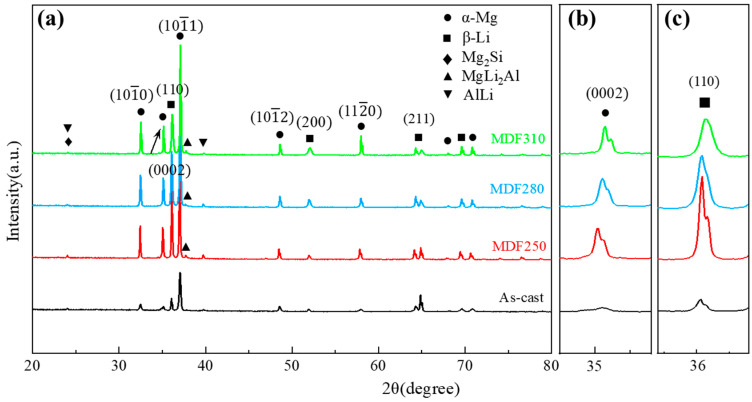
The XRD patterns of the LA83-0.3Si alloy before and after MDF: (**a**) the scanning results of the diffraction angle 20–80°, (**b**) the (0002) plane’s enlarged image of the α phase, and (**c**) the (110) plane’s enlarged image of β phase.

The XRD patterns of the LA83-0.3Si alloy before and after MDF are shown in Figure 2. Two main peaks of α-Mg and β-Li can be seen in the XRD pattern, which confirms that the LA83-0.3Si alloy is composed of α-Mg and β-Li phases. In addition, there are diffraction peaks of Mg_2_Si and AlLi in the alloys, indicating that there are Mg_2_Si and AlLi phases in addition to the α and β phases. After MDF, a new peak of MgLi_2_Al appeared at the position of 2*θ* = 37°, indicating that the MgLi_2_Al phase was precipitated in the alloy after MDF.

Since the α-Mg phase is the hcp structure and the β-Li phase is the bcc structure [20,32,33,34], the main slip surfaces of the two phases are selected to correspond to the diffraction peaks, and the enlarged images are shown in Figure 2b,c. It can be seen that the diffraction peaks of the (0002) plane of the α phase and the (110) plane of the β phase gradually become wider and move to the right. Tan et al. [35] observed a shift in the main XRD diffraction peak of the chromium-based, high-entropy alloy. They concluded that temperature influences the solid solubility of atoms, leading to local lattice distortions and consequently causing the diffraction peak to shift. Wang et al. [36] also found a similar rule in XRD. The diffraction peak angle *θ* of the (0002) plane of the α phase also increases as the forging temperature increases. This is generally attributed to the enhanced solid solubility of Li in Mg with a rising temperature [37]. Li atoms integrate into the α-Mg lattice, inducing lattice distortion and causing a gradual shift in the diffraction peaks. Similarly, the diffraction peak of the (110) plane of the β phase shifts progressively, which is also attributed to atomic dissolution.

**Figure 3:**
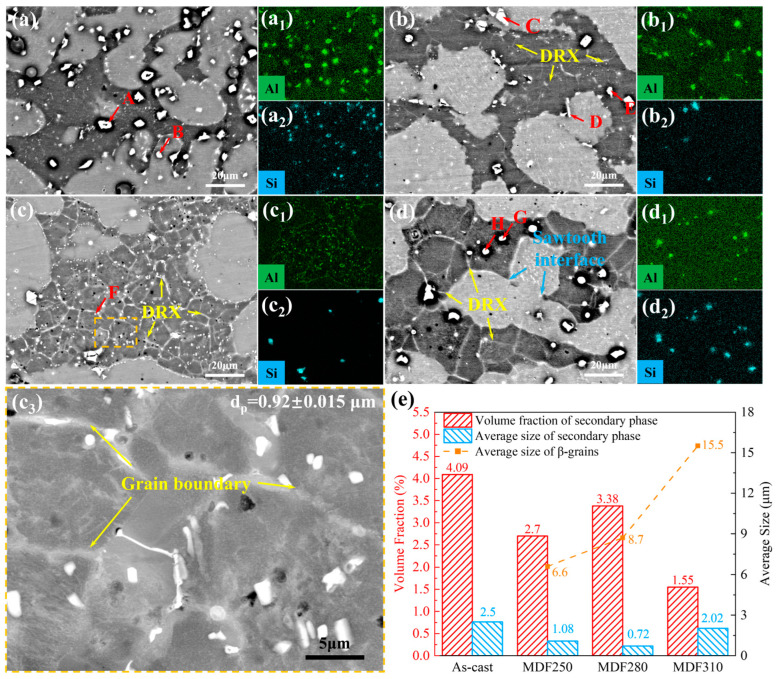
High-magnification BSE images and EDS surface scans of the LA83-0.3Si alloy before and after MDF: (**a**,**a_1_**,**a_2_**) as-cast, (**b**,**b_1_**,**b_2_**) MDF250, (**c**,**c_1_**–**c_3_**) MDF280, (**c_3_**) High-magnification BSE image of the orange region in (**c_2_**), (**d**,**d_1_**,**d_2_**) MDF310, and (**e**) statistical analysis of second-phase particles and β-Li grains in the LA83-0.3Si alloy before and after MDF.

The high-magnification BSE images and EDS surface scan analysis results of the LA83-0.3Si alloy before and after MDF are presented in Figure 3. A summary of the EDS point scan results is provided in Table 1. It should be noted that due to the truncation of the Li spectrum, the EDS system is unable to detect the presence of Li. Consequently, the EDS results do not include data for the Li element [38,39]. From Figure 3a, there are large second phases in both the α and β phases of the as-cast alloy, in which the Al element is enriched in the β-Li phase and the phase boundary forms a spherical or strip phase. As shown in Table 1, the proportion of Al atoms in point A is close to 40%. Based on the XRD patterns and the existing research, it can be inferred that these second phases are AlLi phases [13,40,41]. The Si element is mainly enriched in the α-Mg phase, and the irregular block phase is formed at the phase boundary. The existing research results show that Si reacts with Mg to form the Mg_2_Si phase, which is a high-temperature-stable phase [17,42,43].

In addition, according to the binary phase diagram of the Li-Si alloy [44], when the mass fraction of Si is 0.3%, it will react with Li to form the Li_22_Si_5_ phase. Braga et al. [45] optimized the Li-Si phase diagram and found that the Li_22_Si_5_ phase was an unstable phase from the results of phonon spectroscopy and thermodynamic data. According to the result, combined with the Gibbs free energy of the Mg_2_Si phase, calculated by HSC 6.0, the change curve of the Gibbs free energy of the Li_22_Si_5_ and Mg_2_Si phases formed at 0–600 °C was obtained, as shown in the Supplementary File, Figure S3. The linear fitting of the results shows that the ΔG of the Li_22_Si_5_ phase formed at 720 °C is 4.59 kJ, and the ΔG of the Mg_2_Si phase is −69.35 kJ. We have demonstrated that Mg_2_Si exhibits a significantly more negative ΔG than Li_22_Si_5_, which enables its preferential formation through Si and Mg reactions due to the stronger thermodynamic driving force derived from its lower ΔG value. While the positive ΔG of Li_22_Si_5_ suggests its potential transient existence is influenced by kinetic factors, the system ultimately evolves toward the thermodynamically stable Mg_2_Si phase, a transition that is consistently confirmed by XRD patterns and EDS analytical results.

The microstructure of the LA83-0.3Si alloy after MDF at 250 °C is depicted in Figure 3b. Compared to the as-cast alloy, a few grain boundaries appear in the β phase, indicating recrystallization. Additionally, after MDF, the quantity of the AlLi phase within the β phase is significantly reduced. However, the strip-like AlLi phase at the α/β phase boundary (marked as point D) is retained, and the Mg_2_Si phase remains distributed within the α-Mg (marked as point C). Furthermore, a small-sized phase (marked as point E) was found within the β phase, with an Al atomic proportion of approximately 23%. Based on the XRD results, this particle is identified as the newly formed MgLi_2_Al phase after MDF. As the forging temperature increases, a substantial number of grain boundaries emerge in the β phase, and the number of DRX grains rises, as shown in Figure 3c. At 280 °C, the AlLi phase nearly vanishes, while numerous fine second phases (marked as F points) are observed within the β phase and at the α/β phase boundary, measuring approximately 0.92 μm in size. Point scanning reveals an Al atomic ratio of about 7.7% at the F points. Based on the XRD results, these phases are inferred to be micron-sized MgLi_2_Al phases. At the forging temperature of 310 °C, the significant growth of β-Li grains was observed, which was accompanied by the enhanced spheroidization of the α-Mg phase, along with the inward bending of β phase boundaries that were found to generate more serrated interfaces, as demonstrated in Figure 3d. Compared to the MDF280 alloy, the quantity of the MgLi_2_Al phase was significantly reduced, accompanied by the appearance of larger sized second phases. Figure 3(d_2_) reveals Si element enrichment in some spherical phases. Point scanning at the G and H points indicated the presence of Mg, Al, and Si elements.

Subsequently, a statistical analysis of the second phase and β-Li grains in the LA83-0.3Si alloy before and after MDF was conducted, as shown in Figure 3e. In the as-cast alloy, the AlLi phase is primarily located at the β phase and the α/β phase boundary, while Mg_2_Si is a stable phase distributed within the α phase and the α/β phase boundary. The measured volume fraction is 4.09%, with an average size of approximately 2.5 μm. After MDF, the grain size of β-Li is about 6.6 μm, and both the volume fraction and average size of the second phase decrease, primarily due to the dissolution of a significant number of AlLi phases. In addition, a small number of MgLi_2_Al phases are precipitated in the matrix. This is because the residual Al is still distributed throughout the as-cast alloy, and low-temperature deformation is beneficial to its interaction with the matrix to form a new precipitated phase. The grain size of β-Li grows to 8.7 μm, the AlLi phase completely dissolves, and a substantial amount of MgLi_2_Al phase precipitates as the forging temperature increases. As a consequence, the volume fraction of the secondary phase increases from 2.7% at lower temperatures to 3.38%, while the phase size decreases from 1.46 μm to 1.21 μm. The atomic diffusion velocity accelerates with rising temperatures. Upon the dissolution of the AlLi phase into the β phase, Mg, Li, and Al atoms may segregate at the α/β and β/β interfaces. This segregation not only influences the stability of the β phase interface but also provides a driving force for the nucleation of the metastable MgLi_2_Al phase [46]. The MgLi_2_Al phase is distributed along the grain boundaries, inhibiting grain growth to some extent, resulting in a low growth rate of β-Li grain size. When the forging temperature reaches 310 °C, the average size of β-Li grains is 15.5 μm, indicating that the solid solution behavior of the MgLi_2_Al phase facilitates the migration of β-Li grain boundaries. Additionally, despite the decrease in the volume fraction of the secondary phase, its average size increases, predominantly distributed around Mg_2_Si particles and the grain boundaries of the β phase.

**Figure 4:**
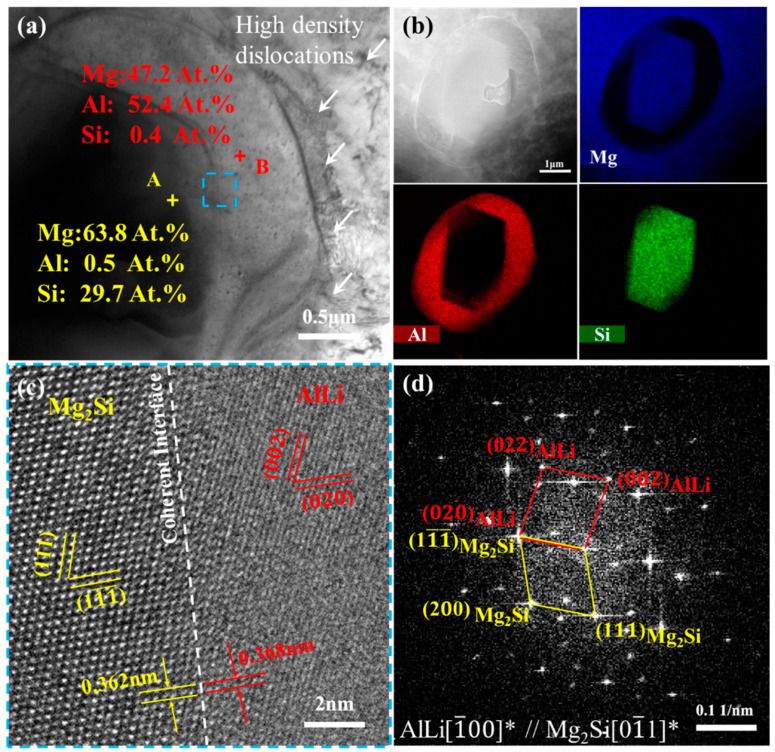
TEM analysis of spherical precipitates of MDF310 alloy: (**a**) bright field (BF) image, (**b**) EDS images of precipitates, (**c**) HRTEM image of blue region in (**a**), (**d**) SAED image corresponding to (**c**). “*” represents the crystal band axis calibrated by the two-phase diffraction spots.

To further determine the precipitated phase composition in the β-Li of the MDF310 alloy, the TEM microstructure observation was carried out, and the results are shown in Figure 4. Figure 4a is the bright field image of the spherical phase, which displays a bright field image of the spherical phase, revealing a block phase within the precipitate, surrounded by high-density dislocations. The surface scan results (Figure 4b) indicate that the spherical phase is predominantly composed of Al, while the internal block phase is enriched with Mg and Si. The atomic ratio of Mg to Al in the spherical phase is approximately 1:1, and the Mg to Si ratio is close to 2:1. Figure 4c presents the lattice image at the two phase interface, with the selected area electron diffraction (SAED) at the interface obtained via Fourier transform, as illustrated in Figure 4d. The interplanar spacing of (020) is 0.368 nm, consistent with the standard interplanar spacing of the AlLi (PDF#65-3017) phase. Meanwhile, the interplanar spacing of ($1\bar{1}\bar{1}$) is 0.362 nm, closely resembling the standard interplanar spacing of the Mg_2_Si (PDF#65-2988) phase. Additionally, a specific crystallographic orientation relationship exists between the AlLi and Mg_2_Si phases: ${\left(020\right)}_{AlLi}$//${\left(1\bar{1}\bar{1}\right)}_{Mg_{2}Si}$, ${\left[\bar{1}00\right]}_{AlLi}$//${\left[0\bar{1}1\right]}_{Mg_{2}Si}$. The interface misfit is calculated using the following formula [47]:

(1) $$\delta =\frac{d{\left(020\right)}_{AlLi}-d{\left(1\bar{1}\bar{1}\right)}_{Mg_{2}Si}}{d{\left(020\right)}_{AlLi}}\times 100\%$$

The calculated misfit value, *δ*, is approximately 1.6%, indicating good atomic matching between ${\left(020\right)}_{\mathrm{AlLi}}$ and ${\left(1\bar{1}\bar{1}\right)}_{Mg_{2}Si}$, which confirms a coherent interface. It is further confirmed that the AlLi phase exists in the form of the Mg_2_Si phase at this temperature.

**Figure 5:**
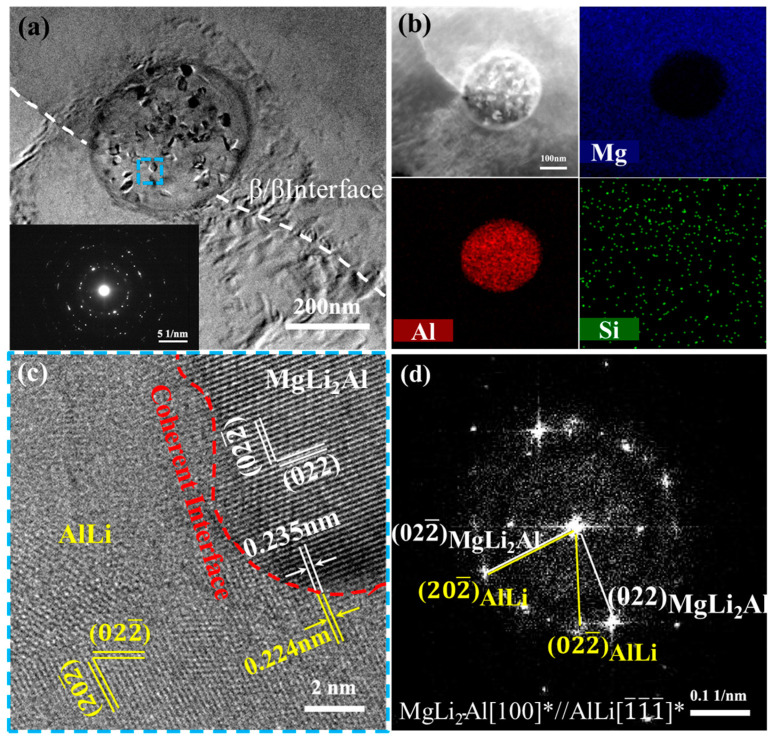
TEM analysis of precipitates at the β-Li grain boundary of MDF310 alloy: (**a**) BF image and FFT image of precipitates, (**b**) EDS images of precipitates, (**c**) HRTEM image of blue region in (**a**), (**d**) SAED image corresponding to (**c**). “*” represents the crystal band axis calibrated by the two-phase diffraction spots.

In addition, Figure 5a is a spherical precipitate at the β-Li grain boundary, and the FFT image shows a ring pattern, indicating that a large number of nanoscale particles are distributed inside. The surface scan results show that it mainly contains the Al element, as shown in Figure 5b. Combined with XRD and the above results, the spherical second phase is the AlLi phase. Figure 5c shows the lattice image at the interface of the nanoscale secondary phase. From the SAED pattern in Figure 5d, the interplanar spacing of (20$\bar{2}$) is 0.224 nm, consistent with the standard interplanar spacing of the AlLi (PDF#65-4215) phase. The interplanar spacing of the nanoscale secondary phase (02$\bar{2}$) is 0.235 nm, closely resembling the standard interplanar spacing of the MgLi_2_Al (PDF#65-5657) phase. In addition, there is a specific crystal orientation relationship between the AlLi phase and the MgLi_2_Al phase: ${\left(02\bar{2}\right)}_{{\mathrm{MgLi}}_{2}\mathrm{Al}}$//${\left(20\bar{2}\right)}_{AlLi}$, ${\left[100\right]}_{{\mathrm{MgLi}}_{2}\mathrm{Al}}$//${\left[\bar{1}\bar{1}\bar{1}\right]}_{\mathrm{AlLi}}$. The lattice mismatch at the two-phase interface is approximately 3.14%. This indicates a coherent interface between (02$\bar{2}$) of the MgLi_2_Al phase and (20$\bar{2}$) of the AlLi phase. It is evident that the AlLi phase is attached to the MgLi_2_Al phase.

Wang et al. [48] demonstrated that the MgLi_2_Al phase is metastable, while the AlLi phase (B2 structure) is stable. Elevated annealing temperatures promote MgLi_2_Al dissolution and AlLi precipitation, which is consistent with our findings. Notably, although second-phase particles typically precipitate along grain boundaries, AlLi phase preferentially nucleates at Mg_2_Si phases. Sun et al. [49] observed that during deformation, a high dislocation density and large orientation gradient region, termed the particle deformation zone (PDZ), forms around SiC_p_. A PDZ with a high dislocation density enhances atomic diffusion, facilitating the formation of precipitated phases. As a stable, hard particle, the Mg_2_Si phase at the phase boundary coarsens with the β phase during deformation. This process induces significant lattice distortion and impedes dislocation movement, leading to high-density dislocation regions near the particles. The existence of these dislocations accelerates the diffusion of Al from the dislocation-poor region adjacent to the β-Li matrix, which in turn promotes the enrichment of solutes near the particles and provides favorable conditions for the precipitation of the AlLi phase on the surface of Mg_2_Si [50]. Due to the coherent interface between the AlLi and Mg_2_Si phases, the AlLi phase readily attaches to the Mg_2_Si phase. The surface scan results in Figure 3(d_1_,d_2_) reveal that in the β phase, the phases enriched with Si elements are enriched with Al elements. Thus, after MDF at 310 °C, the AlLi phase precipitates by adhering to the Mg_2_Si phase.

### 3.2. EBSD Microstructure of LA83-0.3Si Alloy After MDF

**Figure 6:**
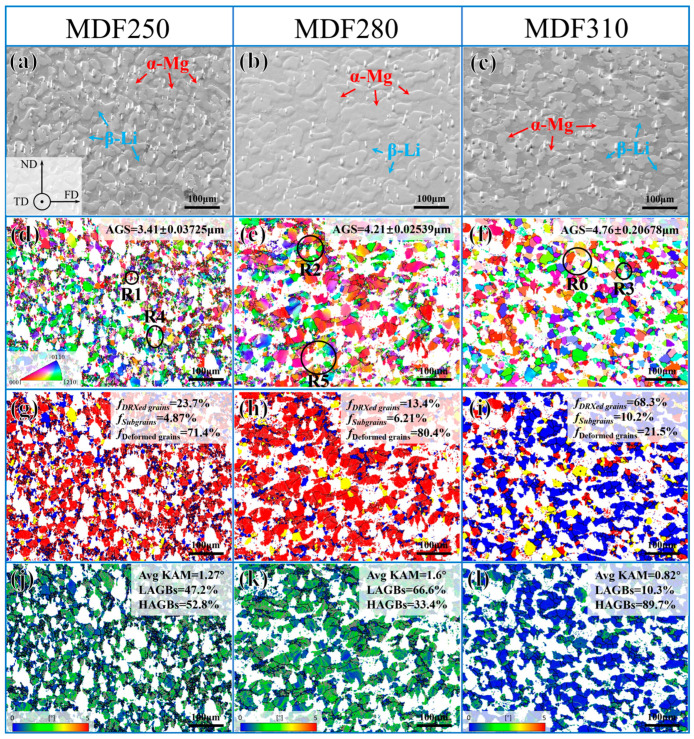
Microstructure of α-Mg phase in LA83-0.3Si alloy after MDF: (**a**–**c**) slectropolished SEM images, (**d**–**f**) IPF maps, (**g**–**i**) recrystallized grain distribution maps (blue represents DRXed grains, yellow indicates sub-grains, and red denotes deformed grains.), (**j**–**l**) KAM maps.

In previous studies, DRX occurs in the β phase following MDF, with DRX grains progressively growing as the forging temperature increases. The α-Mg grains cannot be discerned from the BSE images, so the α phase was characterized using EBSD, as shown in Figure 6. To minimize noise interference, orientation deviation angles less than 2.5° were excluded. Low angle grain boundaries (LAGBs, 2.5° to 15°) are indicated in silver, while high angle grain boundaries (HAGBs, >15°) are marked in black. In the recrystallization distribution images (Figure 6g–i), blue represents DRXed grains, yellow indicates sub-grains, and red denotes deformed grains.

Figure 6a–c show the SEM images of the alloy after electrolytic polishing after MDF. In these images, the protruding phase is identified as the α-Mg phase, while the concave phase is the β-Li phase. Figure 6d reveals that the grain size of α-Mg after MDF is relatively small, with an average size of approximately 3.41 μm. The white area in Figure 6g represents the unresolved β phase-affected zone. Following MDF, the recrystallization fraction of the alloy is about 23.7%, with unrecrystallized (unDRXed) grains comprising 76.3%. Throughout the observation field, deformed grains predominate, and LAGBs account for approximately 47.2%, indicating that DRX has occurred in the α-Mg grains. These DRX grains are continuous, lacking distinct nucleation and growth stages, and can, therefore, be classified as continuous dynamic recrystallization (CDRX) grains [51]. The analysis of the KAM map, shown in Figure 6j, revealed an average KAM value of 1.27°. A significant accumulation of dislocations can be observed in these deformed grains, with a relatively high dislocation density near the Mg_2_Si phase.

A significant number of twisted, deformed grains appear in the IPF map as the forging temperature increases, as shown in Figure 6e. Compared to the MDF250 alloy, the average grain size increases from 3.41 μm to 4.21 μm. With rising forging temperatures, the recrystallization fraction decreases from 23.7% to 13.4%, while the proportion of unDRXed grains increases. Additionally, LAGBs increase from 47.2% to 66.6%. Furthermore, at locations distant from the β phase, DRX grains are distributed along the boundaries of deformed grains and can be classified as CDRX grains. The number of DRX grains at the α/β phase boundary is limited. When the forging temperature reaches 310 °C, fine grains with random orientations predominate throughout the observation field (Figure 6f), with an average grain size of approximately 4.76 μm. As shown in Figure 6i,l, further increasing the forging temperature leads to a rise in the recrystallization fraction from 13.4% to 68.3%, a decrease in the average KAM value from 1.6° to 0.82°, and a significant reduction in the dislocation density. At 310 °C, the deformed grains absorb a substantial number of dislocations, facilitating the continuous transformation of LAGBs into HAGBs (89.7%) and promoting the nucleation and growth of DRX at the phase boundary, indicative of the discontinuous dynamic recrystallization (DDRX) mechanism [52,53].

In summary, the proportion of DRX grains in both the α and β phases increases with rising forging temperatures, indicating a more complete recrystallization process. This enhancement is attributed to higher temperatures facilitating dislocation movement and grain boundary migration, thereby promoting the nucleation and growth of DRX grains [54]. However, the formation of DRX grains in the α-Mg phase is inhibited at 280 °C, primarily due to microstructural evolution. Previous studies have indicated that a reduction in interface energy can impede the DRX process [55]. In the MDF280 alloy, a significant amount of the MgLi_2_Al phase precipitates at the α/β phase boundaries, which decreases the interface energy and obstructs the nucleation pathways for DRX. Consequently, this reduction in interface energy diminishes the driving force for DRX.

**Figure 7:**
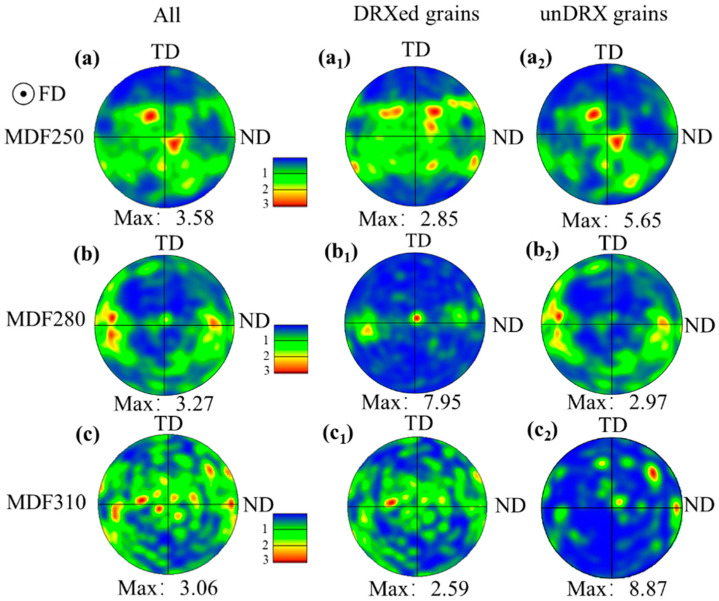
(0002) pole figures of α-Mg grains and their corresponding DRXed and unDRXed grains after MDF in LA83-0.3Si alloy: (**a**,**a_1_**,**a_2_**) MDF250, (**a_1_**) Pole figure of DRXed grains, (**a_2_**) Pole figure of unDRXed grains, (**b,b_1_,b_2_**) MDF280, (**b_1_**) Pole figure of DRXed grains, (**b_2_**) Pole figure of unDRXed grains, (**c**,**c_1_**,**c_2_**) MDF310, (**c_1_**) Pole figure of DRXed grains, (**c_2_**) Pole figure of unDRXed grains.

The (0002) pole figure of the α-Mg phase following MDF of the LA83-0.3Si alloy is presented in Figure 7. The analysis of Figure 7a–c reveals a bimodal texture in the basal pole figure after MDF, with deflections from the FD to the normal direction (ND) of 15.73°, and from FD to both ND and the transverse direction (TD) of 29°. The initial maximum pole density is recorded at 3.58. The texture peak shifts as the forging temperature increases, resulting in a reduce in the maximum pole density to 3.27. Upon reaching 310 °C, multiple randomly distributed texture peaks emerge in the basal pole figure, and the maximum pole density decreases to 3.06.

To further investigate the impact of DRX grains on the basal texture, the texture distribution maps of the DRX and unDRXed grains were analyzed separately. As shown in Figure 7(a_1_,a_2_), the c-axis of the DRXed grains after MDF predominantly deflects towards the FD and the ND to varying extents. In contrast, the unDRXed grains primarily deflect along the FD, with their texture type consistent with that of the entire grain population. The DRX grains reduce the maximum pole density (5.65) of the unDRXed grains, leading to an overall decrease in the texture intensity.

The basal pole figure of the DRX grains exhibits a pronounced peak along the FD as the forging temperature increases. The grain orientation gradually shifts from the FD and ND to being more aligned with the FD, resulting in an increased maximum pole density of 7.95. In contrast, the texture peaks of the unDRXed grains transition from an original bimodal structure to a multi-peak configuration, with peaks at 65°, 67°, and 62°, deflecting from the FD to the TD. The maximum pole density decreases to 2.97, aligning with the texture type of the entire grain population. At 280 °C, the activation of non-basal slip systems during deformation causes the unDRXed grains to rotate, reducing the pole density. When the temperature reaches 310 °C, the DRX grains exhibit a multi-peak texture, with all the grains becoming randomly oriented, significantly reducing the maximum pole density to 2.59. Meanwhile, the orientation of the unDRXed grains becomes concentrated, and the maximum pole density increases from 2.97 to 8.87. Thus, the DRX grains significantly influence the texture type and strength of the overall grain structure.

Therefore, the α-Mg phase in the LA83-0.3Si alloy undergoes DRX after MDF, during which the texture type and intensity distribution of the overall grains are predominantly governed by the high fraction of deformed grains, as evidenced by the pole figure analysis that revealed significantly enhanced basal texture characteristics. The DRX grain fraction is observed to decrease as the forging temperature increases, resulting in an elevation of the maximum pole density value of the texture. Simultaneously, the elevated temperature promotes plastic deformation behavior in grains, which leads to the formation of dispersed texture distribution patterns in pole figures. When the forging temperature reaches 310 °C, a marked increase in the DRX grain fraction is triggered, which plays a dominant role in the texture evolution mechanism of the alloy. The proliferation effect of DRX grains not only facilitates the homogenization of texture distribution, but effectively weakens the texture intensity, ultimately contributing to significant anisotropy reduction in the alloy.

### 3.3. Mechanical Properties of LA83-0.3Si Alloy Before and After MDF

**Figure 8:**
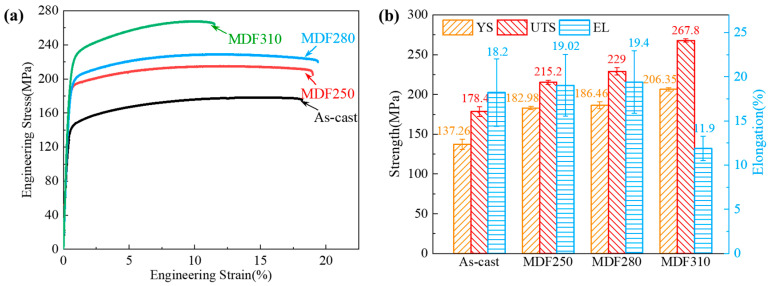
Mechanical properties of LA83-0.3Si alloy before and after MDF: (**a**) stress–strain curves from room-temperature tensile tests, (**b**) bar chart of tensile properties.

Figure 8 is the tensile stress–strain curve and the corresponding mechanical properties histogram of the LA83-0.3Si alloy before and after MDF. The yield strength (YS), ultimate tensile strength (UTS), and elongation (EL) of the as-cast alloy are 137.26 MPa, 187.4 MPa, and 18.2%, respectively. After MDF, the YS, UTS, and EL of the as-cast alloy reach 182.98 MPa, 215.2 MPa, and 19.02%, respectively. With the increase in the forging temperature, the strength and plasticity of the alloy are further improved. When the temperature reaches 310 °C, the YS and UTS values of the MDF310 alloy reach the highest values of 206.35 MPa and 267.8 MPa, which are about 50% higher than those of the as-cast alloy, but the EL decreases from 19.4% to 11.9%.

**Figure 9:**
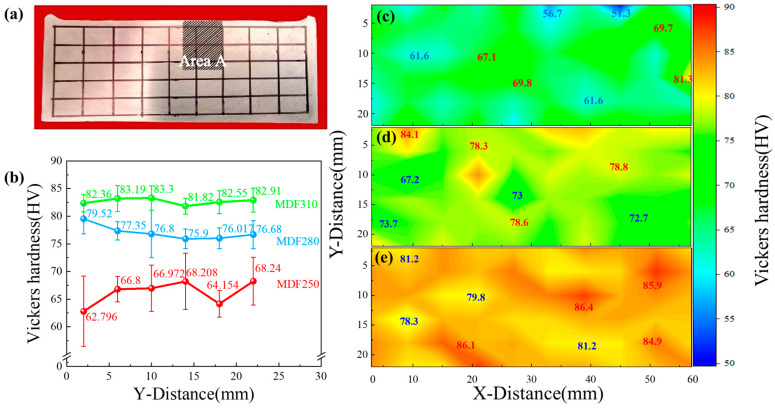
(**a**) Forging surface macrograph. (**b**) Micro Vickers hardness statistics and micro Vickers hardness nephogram: (**c**) MDF250, (**d**) MDF280, (**e**) MDF310.

The microhardness of the LA83-0.3Si alloy is shown in Figure 9. The test position is within a 10 × 6 grid in Figure 9a. The hardness value of each mesh intersection was statistically analyzed, and the hardness change diagram of the forged surface was drawn (Figure 9b). The figure indicates that as the MDF temperature increases, the surface hardness progressively rises. The relative change rate, Δ*δ*, is used to quantify the variation in the average hardness across each row:

(2) $$\Delta \delta =({\bar{HV}}_{\max}-{\bar{HV}}_{\min})/{\bar{HV}}_{\min}\times 100\%$$

The Δ*δ* values for the MDF250, MDF280, and MDF310 alloys are 8.6%, 4.7%, and 1.8%, respectively, indicating a stabilization of the overall hardness from the initial fluctuations. To more intuitively display the hardness distribution, cloud maps are provided in Figure 9c–e.

The forging surface transitions from low-hardness cyan to high-hardness orange as the forging temperature increases. The MDF250 alloy map (Figure 9c) shows dark blue regions with a hardness difference of about 6 HV, attributed to the large AlLi phase. In Figure 9d, the MDF280 alloy map reveals increased surface hardness and reduced variation due to the dissolution of the AlLi phase and the precipitation of the metastable MgLi_2_Al phase. The MDF310 alloy (Figure 9e) exhibits higher hardness, with a substrate hardness difference of approximately 3.7 HV, associated with the stable AlLi phase.

**Figure 10:**
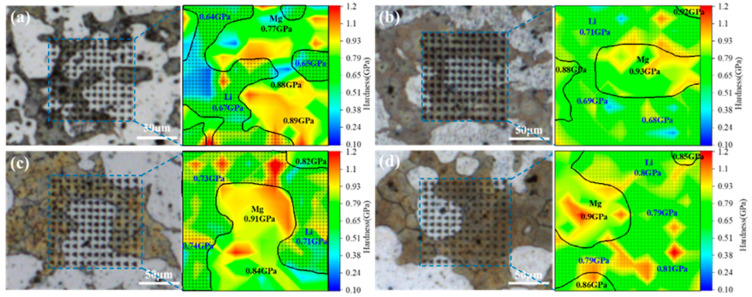
Nanoindentation hardness distribution of LA83-0.3Si alloy before and after MDF: (**a**) as-cast, (**b**) MDF250, (**c**) MDF280, (**d**) MDF310.

The nanoindentation hardness of the α and β phases in the LA83-0.3Si alloy, as depicted in Figure 10, corresponds to the area labeled A in Figure 9a. The α phase and the β phase regions were delineated, and the hardness average values for each region were measured. XPM images indicate that the hardness of the α-Mg phase exceeds that of the β-Li phase, aligning with the existing literature [56]. After MDF, the hardness of both the α and the β phases increased, with a significant disparity between them. As the forging temperature rose, the hardness of the α phase remained relatively constant, while the hardness of the β phase progressively increased. At a forging temperature of 310 °C, the β phase exhibited maximum hardness, and the difference in hardness between the α and β phases was minimized. In conjunction with previous results, these results further confirm that the enhanced hardness of the β phase is attributed to the dissolution of solid solution atoms.

### 3.4. Fracture Behavior of LA83-0.3Si Alloy Before and After MDF

**Figure 11:**
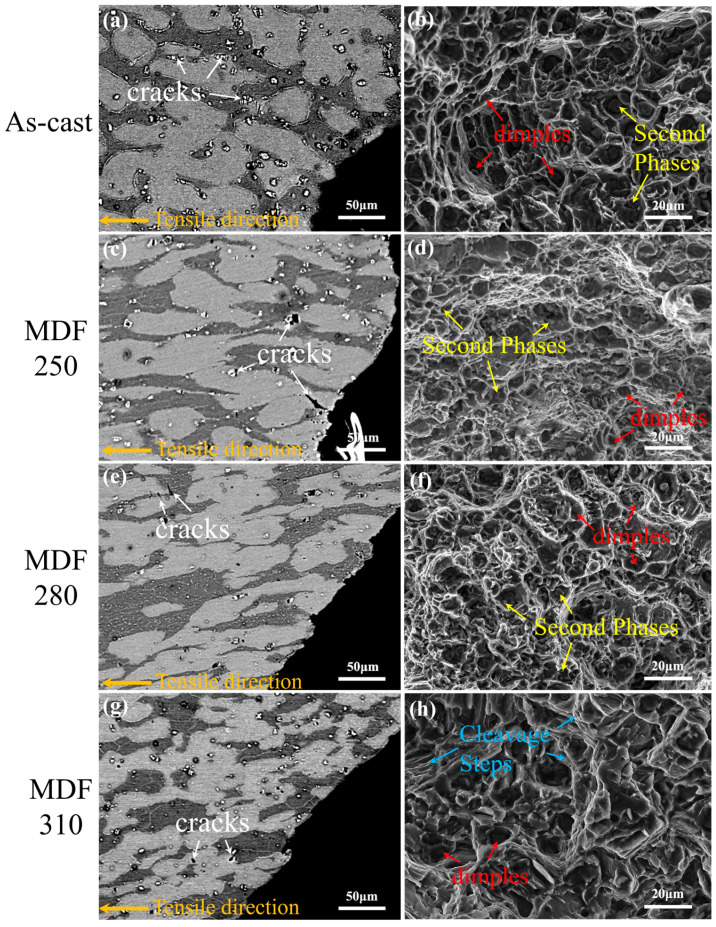
The microstructure of the alloy surface after tensile fracture in various states: (**a**,**c**,**e**,**g**) BSE images of the side fracture, (**b**,**d**,**f**,**h**) SEM images of the front fracture.

The fracture morphology of the LA83-0.3Si alloy, both before and after MDF, is illustrated in Figure 11. In the as-cast alloy (Figure 11a,b), the short, rod-like β phase in the side fracture is elongated, and pits formed by the debonding of large AlLi phases are evident. Additionally, microcracks predominantly appear in the Mg_2_Si phase, with none observed in the matrix. The front fracture of the as-cast alloy exhibits deep and wide dimples (indicated by red marks), with second-phase particles dispersed at the base of these dimples (indicated by yellow marks). Consequently, the tensile fracture mode of the as-cast alloy is characterized as a ductile fracture.

Figure 11c,d depict the side and front fracture microstructures of the MDF250 alloy. After MDF, the β phase remains elongated along the FD direction, with small pits resulting from the debonding of second-phase particles at the fracture. Microcracks extend from the Mg_2_Si phase into the α phase, while no microcracks are detected in the β phase. The front fracture features deep and wide dimples, with second-phase particles scattered at their base.

When the forging temperature rises, the fracture edge becomes relatively flat, with only minimal particle debonding observed. Additionally, cracks are noted in the β phase with a high aspect ratio. Numerous shallow and dense dimples appear on the front fracture, with fine second-phase particles at their base, indicating characteristics of ductile fractures. At 310 °C, the side fracture’s edge becomes smooth, lacking pits from particle debonding, and deep, long cracks emerge in both the α and β phases, as shown in Figure 11g,h. Compared to the MDF280 alloy, the MDF310 alloy exhibits fewer dimples on the front fracture, with smooth fracture surfaces and cleavage steps (indicated by blue marks), characteristic of a ductile–brittle mixed fracture.

The analysis indicates that during the tensile process of the as-cast alloy, the Mg_2_Si phase hinders the deformation of the α-Mg matrix, leading to a stress concentration near the phase and initiating cracks. When the cracks extend into the matrix, the stress concentration is somewhat alleviated, inhibiting further crack propagation. After MDF, dislocation movement in the α phase relies on basal slip, while the non-basal slip system in the β phase is activated, resulting in preferential plastic deformation. Due to the different degrees of deformation of the two phases in the matrix, stress concentration is more likely to occur inside the matrix, which is difficult to deform [57]. Liu et al. [58] observed that coordinated deformation between the matrix alloy and the composite material’s soft and hard layers can alleviate stress concentration during tension. The interface layer effectively passivates crack tips through plastic deformation, increasing resistance to crack propagation. The nanoindentation results reveal significant differences in the hardness values of the α and β phases, indicating that the β phase effectively transfers stress, thereby alleviating the stress concentration in the α phase. Consequently, during tensile deformation, microcracks propagate from the Si phase into the α phase matrix and halt near the β phase. As the forging temperature increases, the coordinated deformation capability of the α/β phases diminishes due to the increased hardness of the β phase. Thus, during tensile deformation, microcracks extend from the α phase into the β phase, which undergoes greater deformation. At 310 °C, the β phase exhibits maximum hardness, and the deformation coordination between the α and β phases is minimized. Therefore, during tension, the β phase’s ability to mitigate stress concentration in the α phase is reduced, facilitating crack propagation.

## 4. Discussion

### 4.1. DRX Behavior of LA83-0.3Si Alloy

In recent years, extensive research has focused on the recrystallization behavior of magnesium alloys. Besides the effects of the deformation temperature and strain, the second phase plays a crucial role. Fan et al. [59] explored the DRX behavior of Mg grains influenced by hard particles, demonstrating local lattice rotation near these particles. During deformation, numerous LAGBs in the PDZ transform into HAGBs through lattice rotation. As a stable phase within the matrix, the Mg_2_Si phase significantly affects the DRX behavior of α-Mg.

**Figure 12:**
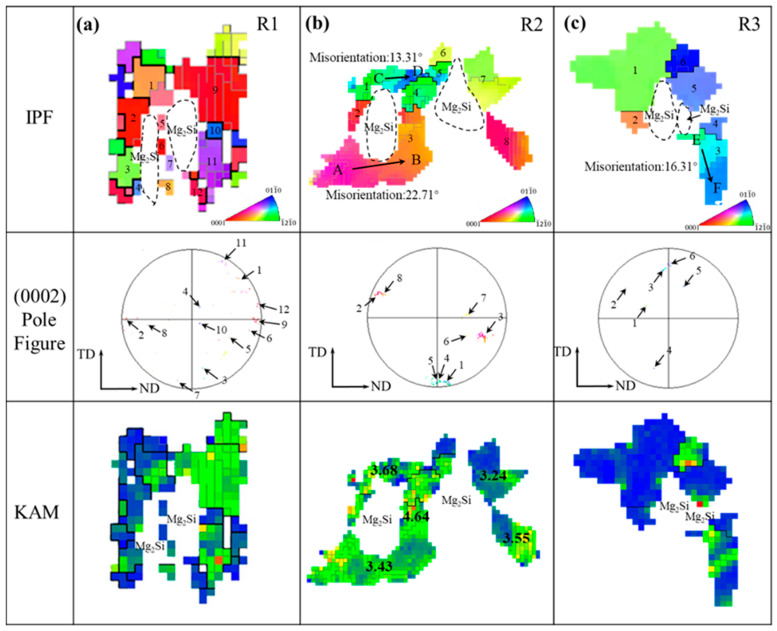
α-Mg grains near Mg_2_Si phase: (**a**) R1 region, (**b**) R2 region, (**c**) R3 region.

The enlarged images of regions R1, R2, and R3 in Figure 6d–f are presented in Figure 12. In the MDF250 alloy, based on the DRX results in Figure 6g, fine DRXed grains are observed around the Mg_2_Si in the R1 region, labeled as 4, 5, 6, 10, and 12, while the remaining are unDRXed grains, as shown in Figure 12a. The formation of these fine DRXed grains around the Mg_2_Si phase is attributed to the rotation of deformed grains around Mg_2_Si during the MDF process, with deformation energy accumulating at the interface. This energy facilitates the transformation of LAGBs to HAGBs in the PDZ, providing the necessary driving force for grain boundary migration [60]. Additionally, finer deformed grains (7, 8) were observed near the Mg_2_Si phase, with a random orientation distribution in the basal pole figure, indicating that Mg_2_Si promotes grain refinement through DRX. The KAM map shows that the stored energy in the grains on the right (9–12) is higher than in those on the left (1–4), suggesting that the Mg_2_Si phase impedes α-Mg deformation, causing an uneven stress distribution on both sides of the particles. The PDZ influence is more pronounced on the right side, resulting in a greater number of newly formed DRX grains.

No DRXed grains were observed near the particles with the increasing forging temperature, as shown in Figure 12b. In the IPF map, the orientation differences along the AB and CD directions were measured to be 22.7° and 13.3°, respectively, further confirming that the rise in temperature facilitates the lattice rotation of the α-Mg phase around the Mg_2_Si particles. In the texture distribution diagram, the adjacent deformed grains exhibit a more concentrated orientation. Additionally, the KAM value at the particle intersection area reaches a maximum of 4.64°. The dislocation density near the particles becomes more uniform as the temperature increases and the stress concentration is alleviated. This phenomenon demonstrates that the coordinated deformation between the Mg_2_Si phase and the α-Mg phase is enhanced, leading to an increased degree of lattice rotation in the grains surrounding the particles. The deformation energy stored during this process primarily provides the driving force for CDRX and the precipitation of the MgLi_2_Al phase [59]. When the forging temperature is increased to 310 °C, the deformed grains near the Mg_2_Si phase are replaced by DRXed grains (marked as 1, 2, 4, and 5). The orientation of these newly formed DRXed grains is randomly distributed, and the dislocation density near the particles is reduced. This indicates that the elevated temperature accelerates the migration rate of sub-grain boundaries, thereby increasing the dislocation orientation gradient of the PDZ and leading to the nucleation of more DRXed grains along the particle surfaces [60].

**Figure 13:**
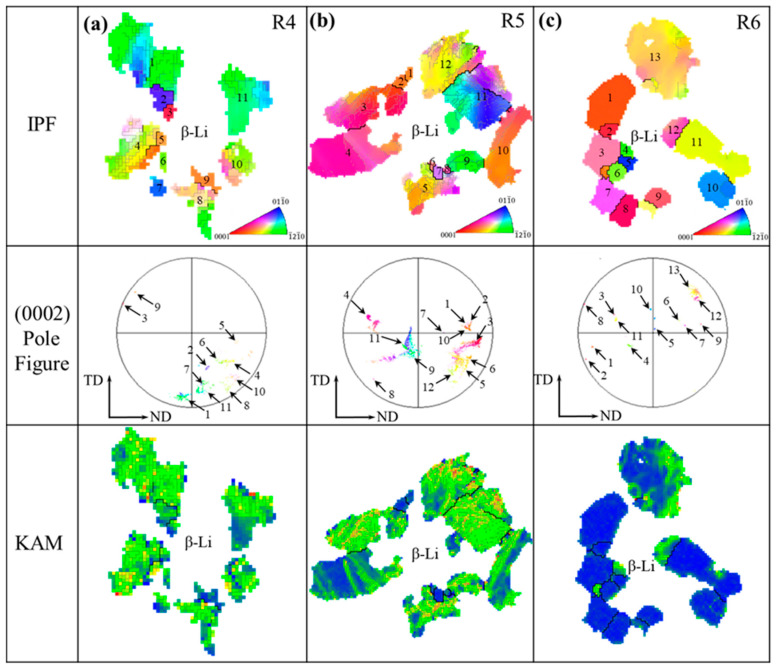
α-Mg grains near the α/β phase boundary: (**a**) R4 region, (**b**) R5 region, (**c**) R6 region.

As previously discussed, increasing the forging temperature results in a progressive increase in the hardness of the β phase. This intrinsic hardening of the β phase significantly influences the DRX behavior of α-Mg. To further analyze the β phase, regions R4, R5, and R6 from Figure 6d–f were selected, with the enlarged diagrams presented in Figure 13.

Figure 13a presents the IPF, basal pole figure, and KAM map for the grains in the R4 region. After MDF, deformed grains are observed around the α/β phase boundary, with their orientations primarily along the TD and ND, resulting in a concentrated texture distribution. The KAM map reveals high-density dislocations near the phase boundary, with a uniform distribution. Due to the greater deformability of the β phase compared to the α phase, the disparity in the deformation capacities leads to a stress concentration at the α/β phase boundary during the MDF process. The β phase, acting as a soft phase, effectively transfers dislocations, alleviating the stress concentration. Some dislocations are consumed by the recrystallization of the β phase, while the remainder is stored in the deformed grains, providing a driving force for the precipitation of CDRX and MgLi_2_Al phases.

The analysis of the R5 region reveals that with the increasing forging temperature, fine DRXed grains (marked as 1, 2, 6, 7, 8) emerge near the phase boundary, as shown in Figure 13b. Notably, DRXed grains 1 and 2 share the same orientation as deformed grain 3, while DRX grain 6 shares the orientation with deformed grain 5. It is generally accepted that the orientation of new CDRX grains closely resembles that of adjacent parent grains [51]. These DRXed grains preferentially form within original grains, with minimal interface migration, resulting in a relatively uniform microstructural transformation.

When the forging temperature increases to 310 °C, the R6 region is mainly composed of deformed grains (3, 4, 12, 13) and DRXed grains. The orientation of newly formed DRXed grains in the basal pole figure is randomly distributed. It is generally believed that most of the DDRXed grains are randomly distributed and not preferentially selected with the parent grains [61,62], and the size of these DDRXed grains varies greatly. Therefore, the DDRX process involves the formation of new DRXed grains (5, 6, 11) by nucleating on the serrated boundaries of parent grains (3, 4, 12) and allowing these nuclei to grow. The KAM map reveals that at elevated temperatures, the number of LAGBs within the grains decreases, and the dislocation density at the α/β phase boundary is significantly reduced, although dislocation accumulation areas persist. It indicates that the increased temperature weakens the coordinated deformation between the α and β phases, with the hardening of the β phase becoming a key factor impeding the deformation of the α phase. It results in uneven stress distribution within the α phase during deformation, forming a PDZ similar to that around the hard particles. Additionally, the dissolution of the MgLi_2_Al phase at the α/β boundary reduces its dislocation pinning effect. Consequently, the orientation difference gradient in the PDZ region near the β phase broadens, increasing the driving force for DRX.

In summary, the increase in the forging temperature is demonstrated to enhance the coordinated deformation between the Mg_2_Si phases and α-Mg. At lower temperatures, the DRX mechanism of the α-Mg adjacent to the Mg_2_Si phases is dominated by the DDRX mechanism, which is initially weakened and subsequently strengthened near particles as temperature rises. After MDF, substantial deformation-stored energy is accumulated within the LA83-0.3Si alloy. Due to the relatively low temperature, this stored energy is fully exploited as the driving force for DRX, during which dislocations in deformed grains are progressively consumed, enabling the transformation of LAGBs into HAGBs, thereby exhibiting CDRX characteristics. The interfacial precipitates occupy potential DRX nucleation sites with the elevation of the forging temperature, resulting in the weakening of the CDRX mechanism. At 310 °C, sufficient thermal activation energy is provided to promote the extensive dissolution of Al-rich phases, through which rapid grain boundary migration is facilitated. This leads to continuous bulging and the subsequent engulfment of deformed grains by newly formed grains, manifesting DDRX behavior. Additionally, the hardening effect of β-Li phases is confirmed to promote both the nucleation and growth of DRX grains, thereby further enhancing DDRX progression.

### 4.2. Work Hardening and Work Softening Behavior Under the Action of α/β Dual Phase

In general, work hardening occurs during the plastic deformation of metals, accompanied by dynamic work softening processes [63]. The work hardening behavior is influenced by dislocation density, while work softening behavior is closely related to the dislocation recovery rate [64]. As described in Section 3.3, MDF enhances the strength and ductility of the LA83-0.3Si alloy. Both YS and the UTS improve significantly with the increasing forging temperature, whereas EL decreases. Additionally, Section 3.4 discusses how the fracture behavior of the alloy, before and after MDF, is related to the coordinated deformation between the α and β phases. Therefore, based on the room temperature tensile test results of the LA83-0.3Si alloy, the effects of MDF are analyzed as follows.

**Figure 14:**
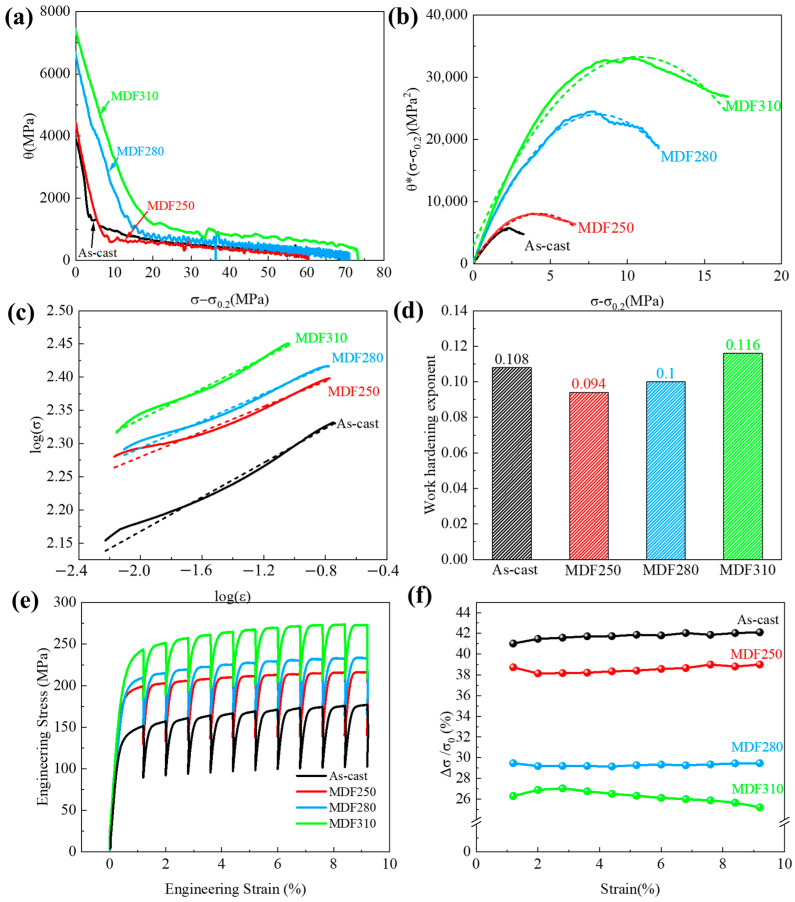
Work hardening and work softening behavior of LA83-0.3Si alloy before and after MDF: (**a**) work hardening rate curve, (**b**) *θ*$\left(\sigma -\sigma_{0.2}\right)$ and $\left(\sigma -\sigma_{0.2}\right)$ relationship curve (the dotted lines represent the corresponding fitted curves), (**c**) true stress–strain logarithmic curve, (**d**) work hardening index, (**e**) cyclic stress relaxation curve, (**f**) work softening rate curve.

The work hardening ability of the alloy is characterized by the work hardening rate, *θ*. A higher *θ* value indicates a stronger work hardening capability. The Kocks–Mecking model effectively describes the relationship between the work hardening rate (*θ*) and the net flow stress $\left(\sigma -\sigma_{0.2}\right)$. The work hardening rate, *θ*, is expressed as follows [65]:

(3) $$\theta =\frac{d\sigma }{d\varepsilon }$$

where, *σ* and *ε* represent the true stress and true strain, respectively. Figure 14a illustrates the variation of *θ* with $\left(\sigma -\sigma_{0.2}\right)$ for the LA83-0.3Si alloy before and after MDF. The diagram reveals that the alloy’s work hardening behavior primarily consists of two stages, that is, a rapid decrease in the strain hardening rate and a stable fluctuation stage, which correspond to the stage of dynamic recovery (Stage III) and the stage of extensive strain hardening (Stage IV) in the Kocks–Mecking model, respectively [66]. Following yielding, all alloys enter the dynamic recovery stage, where the work hardening rate decreases linearly with the increasing strain due to the activation of dislocation cross-slip and the onset of dynamic recovery [67]. During Stage III, the as-cast alloy exhibits the lowest work hardening rate, with the *θ* value decreasing most rapidly as the flow stress increases. In contrast, the work hardening rate of the alloy after MDF increases with the temperature, and the rate of decrease in the *θ* value diminishes as temperature rises.

To further analyze the effect of temperature on the dynamic recovery stage of the LA83-0.3Si alloy, the model established by Lukac et al. [68] was used to analyze the change in the dislocation density with *ρ* and strain *γ* in the alloy before and after MDF. The mathematical expression is as follows:

(4) $$\frac{d\rho }{d\gamma }=k+k_{1}\rho^{1/2}-k_{2}\rho -k_{3}\rho^{2}$$

where $k=\frac{1}{b\times d}$, *b* is the Burgers vector, and *d* is the distance between obstacles that impede dislocation movement, such as grain boundaries, second phase particles, and the matrix phase. The constant *k*_1_ is related to the interaction between dislocations in the material, while *k*_2_ and *k*_3_ pertain to dynamic recovery mechanisms due to dislocation cross-slip and climb, respectively. The literature indicates [69] that the relationship between *dρ*/*dγ* and $\rho^{1/2}$ can be described by the relationship between *θ*$\left(\sigma -\sigma_{0.2}\right)$ and $\left(\sigma -\sigma_{0.2}\right)$, which can be expressed as follows:

(5) $$\theta \left(\sigma -\sigma_{0.2}\right)=k+k_{1}\left(\sigma -\sigma_{0.2}\right)-k_{2}{\left(\sigma -\sigma_{0.2}\right)}^{2}-k_{3}{\left(\sigma -\sigma_{0.2}\right)}^{4}$$

Because the tensile test is carried out at room temperature, for the magnesium alloy, dislocation climbing is difficult to activate; therefore, *k*_3_ is 0. Subsequently, the $\theta \left(\sigma -\sigma_{0.2}\right)$ and $\left(\sigma -\sigma_{0.2}\right)$ relationship curves of the alloys in different states were drawn, as shown in Figure 14b. The shape of the curve is similar to a parabola. In the early stage of plastic deformation (Stage III), with the increase in the flow stress, the dislocation density increases first and then decreases. The polynomial fitting of the curve was carried out to obtain the values of each parameter, and the fitting results are shown in Table 2.

As illustrated in Table 2, increasing the forging temperature results in a continuous rise in the *k* and *k*_1_ values of the LA83-0.3Si alloy, while the *k*_2_ value consistently decreases. It indicates that higher forging temperatures accelerate dislocation accumulation within the alloy, enhance dislocation interactions, and reduce the recovery efficiency due to dislocation cross-slip. Generally, microstructural changes influence the dislocation density, with the grain size and second-phase particles being the primary factors affecting it [70,71]. Sohrabi et al. [72] demonstrated that an increase in the grain size of magnesium alloys enhances work hardening. Fan et al. [65] also obtained similar results when studying the work hardening behavior of magnesium matrix composites. As shown in the Supplementary File, Figure S4a illustrates a dislocation pile-up at the grain boundaries of β-Li grains. As described in Section 3.2, as the forging temperature increases, the grain size of the α and β phases increases, and the number of grain boundaries decreases, resulting in a weakened absorption of dislocations and the easier pile-up of dislocations near the grain boundaries. Furthermore, larger grains provide more space for dislocations to move and accumulate, enhancing dislocation interactions and strengthening the work hardening effect. Additionally, second-phase particles significantly impede dislocation movement. Zhang et al. [73] observed that an increased volume fraction of the second phase elevates the material’s work hardening rate. Section 3.1 indicates that forging temperature influences the evolution of the second phase, with a decrease in the AlLi and MgLi_2_Al phases as temperature rises. It suggests that while the second phase’s impact on work hardening is limited, solid solution atoms play a crucial role in influencing the work hardening effect. Tang et al. [74] found that increasing the annealing temperature enhances the mechanical properties of the Mg-8Li-3Al-2Zn-0.2Y alloy, demonstrating solid solution strengthening in the β phase. Wang et al. [48] reported similar findings for the Mg-10Li-3Al-3Zn alloy. As previously mentioned, the AlLi phase dissolves into the β phase following MDF. At 310 °C, a substantial amount of the MgLi_2_Al phase also dissolves into the β phase. This dissolution causes significant lattice distortion, increasing dislocation motion resistance and enhancing dislocation interactions, thereby strengthening the work hardening effect.

In addition, Zhang et al. [75]. suggested that the interface between soft and hard phases can impede dislocation movement. Based on the nanoindentation results and the findings from Section 4.1, it was concluded that as the forging temperature increases, the number of solute atoms in the β phase rises, leading to its gradual hardening and a reduction in the coordinated deformation capacity of the α/β phases. During tensile testing, when the β phase acts as a soft phase, it primarily facilitates dislocation transfer, improving the distribution of dislocations in the α phase. However, as the forging temperature rises, the β phase becomes harder, hindering α phase deformation and reducing its ability to transfer dislocations. It results in increased dislocation accumulation at phase boundaries and heightened dislocation interactions. Particularly at 310 °C, the volume fraction of the β phase reaches 38.2%, significantly increasing the geometric barrier to dislocation slip. Consequently, the MDF310 alloy, with the highest β phase content, exhibits a stronger hindrance to dislocation movement and a higher work hardening rate compared to other alloys.

In Stage III, the rate of decrease in *θ* is associated with the dynamic recovery of dislocations [64]. Generally, materials with a high dislocation density exhibit a greater likelihood of cross-slip, leading to an increased dynamic recovery rate of dislocations [65,76]. For the LA83-0.3Si dual-phase alloy, as the forging temperature rises, the work hardening rate of the alloy consistently increases, enhancing dislocation interactions. However, Figure 14a clearly demonstrates that the decrease rate of *θ* slows with the rising temperature, and the work hardening rate of the MDF310 alloy is slower compared to other alloys. Analyzing the variation in the *k*_2_ value reveals that with the increasing forging temperature, the ability of dislocations to cross-slip diminishes, resulting in a reduced dynamic recovery rate, attributed to the coordinated deformation between the α and β phases. After MDF, the β phase facilitates dislocation transfer, leading to a uniform stress distribution in the α phase, an enhanced cross-slip ability, and an increased dynamic recovery rate of dislocations. As the forging temperature rises, the β phase progressively hardens, accelerating the proliferation rate of internal dislocations. However, solid solution atoms within the β phase and a high-density dislocation pile-up at the α/β phase boundary impede the dislocation movement in the β phase. Figure S4b illustrates fewer dislocations within the α-Mg phase, but the β-Li phase exhibits a high dislocation density, with accumulation at the α/β phase boundary. Thus, the increasing temperature weakens the β phase’s ability to transfer dislocations, resulting in a reduced dynamic recovery rate.

Subsequently, the work hardening behavior of the LA83-0.3Si alloy, both before and after MDF, was analyzed during Stage IV. The work hardening index (*n*) of the alloy was calculated as follows [75]:

(6) $$n=\frac{lg\sigma }{lg\varepsilon }$$

where *σ* and *ε* represent true stress and true strain, respectively. The fitting curve and the calculated *n* values are shown in Figure 14c,d. The results indicate that the work hardening index, *n*, for the as-cast alloy, MDF250 alloy, MDF280 alloy, and MDF310 alloy after yielding are 0.108, 0.094, 0.1, and 0.116, respectively, demonstrating a gradual increasing trend. Zhao et al. [77] noted that the work hardening behavior during plastic deformation is positively correlated with the dislocation density. The results show that the work hardening index, *n*, of the alloy after MDF increases with the rising temperature. Therefore, the increase in the grain size of the α and β phases, the solid solution of the AlLi and MgLi_2_Al phases, and the reduced coordinated deformation ability between α and β significantly contribute to the increase in the work hardening index, *n*, of the LA83-0.3Si alloy after MDF.

The work hardening behavior of the alloy after MDF was analyzed previously, and its dynamic work softening behavior will be discussed. Figure 14a is the stress–strain curve of the alloy before and after MDF in the cyclic stress relaxation test. It can be seen from the curve that in each cycle, when the strain is constant, the true stress of the alloy decreases rapidly. It is due to the rearrangement of dislocations to low-energy structures and the activation of grain boundary sliding. These mechanisms trigger the work softening of the alloy [78], so the work softening behavior can be reflected by Δ*σ*. The expression is

(7) $$\Delta \sigma =\sigma_{0}-\sigma_{t}$$

where, *σ*_*t*_ represents the stress value of the alloy at the end of each cycle, and *σ*_0_ is the stress value at the beginning of stress relaxation. The ratio of Δσ to *σ*_0_ is then calculated, and the relative reduction rate curve for each cyclic stress, that is, the work softening rate curve, is shown in Figure 14b. Under the same strain, the as-cast alloy exhibits a high work softening rate of approximately 42%, whereas the MDF310 alloy shows a rate of about 25%. As the forging temperature increases, the work softening rate gradually decreases, aligning with the previous trend observed in the *k*_2_ value. Previous studies have indicated that the significant storage from work hardening provides an effective driving force for subsequent work softening [63]. For the dual-phase LA83-0.3Si alloy, changes in microstructure must be considered when evaluating its work softening behavior.

Shi et al. [64] found that the work softening effect of the material in the in situ tensile test was affected by the grain size. The relationship between the grain size (*d*) and the recovery time (*τ*) of the absorbed dislocation is

(8) $$\tau =\frac{\rho bd}{\alpha \dot{\varepsilon }}$$

where *ρ* is the dislocation density, *b* is the Burgers vector, *α* is a constant, and $\dot{\varepsilon }$ is the strain. Consequently, when the strain is held constant, the recovery time of dislocations is proportional to the grain size. In this research, as the forging temperature rises, the grain size of the α and β phases progressively enlarges, leading to longer dislocation recovery times and a reduced work softening rate. Therefore, the influence of grain boundary migration on the material’s work softening behavior is significant and cannot be overlooked [79,80]. Furthermore, as detailed in Section 3.1, a substantial quantity of MgLi_2_Al phases precipitates from the β phase after MDF. Upon further increasing the temperature to 310 °C, AlLi precipitates emerge within the β phase, impeding dislocation motion and thereby diminishing the alloy’s work softening effect. The obstruction effect of solid solution atoms on dislocation movement is analogous to that of the second phase, influencing the alloy’s work softening behavior.

In the context of the alloy’s work softening behavior, the plastic deformation of the β phase plays a crucial role. As described in Section 3.4, plastic deformation occurs in the β phase during tensile testing at room temperature due to the activation of dislocation slip. The plastic deformation capacity of the β phase diminishes with the increasing forging temperatures, leading to a reduced ability for coordinated deformation between the α and β phases, which hinders the dislocation transfer. Therefore, in the MDF310 alloy, due to the hardening behavior of the β phase, the dislocation movement is inhibited, resulting in a higher dislocation density in the alloy, which in turn reduces the driving force in the work softening process. Compared with other temperature MDF alloys, the work softening rate is significantly reduced.

## 5. Conclusions

This work analyzed the microstructural evolution of the LA83-0.3Si dual-phase alloy during multi-directional forging (MDF) under temperature control. The DRX behavior of LA83-0.3Si dual-phase alloy was investigated, and the work hardening and work softening behavior under the action of the α/β dual-phase was discussed. The main conclusions are as follows:

(1) The LA83-0.3Si alloy, after being subjected to MDF, was characterized by a DRX mechanism of the α phase that was dominated by CDRX and DDRX. With the increase in the forging temperature, the DRX mechanism was gradually transformed into DDRX. The elevated temperature was found to weaken the ability of coordinated deformation between the α/β dual phases, while the hardening behavior of the β-Li phase was observed to provide nucleation pathways for the DRX process.

(2) The basal texture of the α phase was transformed from a bimodal to a multimodal distribution, which was accompanied by a progressive reduction in the maximum pole density as the forging temperature was elevated. The overall texture strength was found to be influenced by dynamically recrystallized grains through grain boundary migration. During high-temperature MDF processing, the AlLi phase was stabilized through the encapsulation of the dispersed particles, resulting in enhanced structural stability and the improved anisotropy of the alloy.

(3) The LA83-0.3Si alloy, after being processed by MDF, was observed to exhibit improved strength and plasticity. With the increase in the forging temperature, its strength was enhanced, while the elongation remained largely unchanged. When the temperature was elevated to 310 °C, the strength of the alloy was significantly increased, and the elongation was reduced, with the UTS and EL reaching 267.8 MPa and 11.9%, respectively. This phenomenon was attributed to the hardening behavior that was induced by the β phase.

(4) The alloy demonstrated a pronounced work hardening effect as the forging temperature increased It was attributed to the strengthening contributions from the grain sizes of the α and β phases, solid solution atoms, and the α/β phase interface. The hardening of the β phase reduced the alloy’s work softening capability.

## Figures and Tables

**Table 1:** EDS point scan results of LA83-0.3Si alloy before and after MDF.

Positions	Mg (at.%)	Al (at.%)	Si (at.%)
A	57.6	42.3	0.1
B	84.5	1.5	14
C	64.7	4.2	31.2
D	80.6	19.4	0.0
E	77.2	22.8	0.0
F	92.2	7.7	0.0
G	77.4	13.8	8.8
H	81.0	12.5	6.4

**Table 2:** Fitting parameter values of work hardening of LA83-0.3Si alloy before and after MDF.

Materials	k	k_1_	k_2_	R^2^
As-cast	117	3182	506	0.999
MDF250	666	3437	400	0.978
MDF280	1041	5463	337	0.992
MDF310	2932	5750	271	0.976

## Data Availability

The original contributions presented in this study are included in the article/Supplementary Material. Further inquiries can be directed to the corresponding authors.

## Supplementary Materials

The following supporting information can be downloaded at https://www.mdpi.com/article/10.3390/ma18081829/s1. Figure S1. BSE and EDS characterization of Al-27Si master alloy: eutectic Al-Si matrix and blocky pure Si phase with 3 μm average size. Figure S2. LA83-0.3Si alloy preparation and processing reference diagram: (a) vacuum resistance heating melting equipment structure diagram, (b) MDF principle diagram, (c) MDF microstructure observation surface and tensile sample selection position map. Figure S3. The change of Gibbs free energy of Li_22_Si_5_ and Mg_2_Si phase formed at 0–600 °C. Figure S4. TEM microstructure of α/β phase boundary in MDF310 alloy.
