# Supplementary material for: Effect of Multi-Directional Forging on Microstructure and Mechanical Properties of Dual-Phase Mg-8Li-3Al-0.3Si Alloy
Source: Materials (Basel). 2025 Apr 16;18(8):1829. doi: 10.3390/ma18081829 (PMC12029033; doi:10.3390/ma18081829)
Supplement: Supplementary file 1 [file materials-18-01829-s001.zip › materials-3555489-supplementary.pdf]

Supplementary Materials for

Effect of Multi-directional Forging on Microstructure and Mechanical Properties of Dual-phase Mg-8Li-3Al-0.3Si Alloy

Pengcheng Tian, Cuiju Wang *, Kaibo Nie, Yaniu Li and Kunkun Deng *

Shanxi Key Laboratory of Magnesium Matrix Materials, College of Materials Science and Engineering, Taiyuan University of Technology, Taiyuan 030024, niekaibo@tyut.edu.cn (K.N.);
* Correspondence: wangcuiju@tyut.edu.cn (C.W.); dengkunkun@tyut.edu.cn (K.D.)

Supplementary 1: BSE and EDS characterization of Al-27Si master alloy

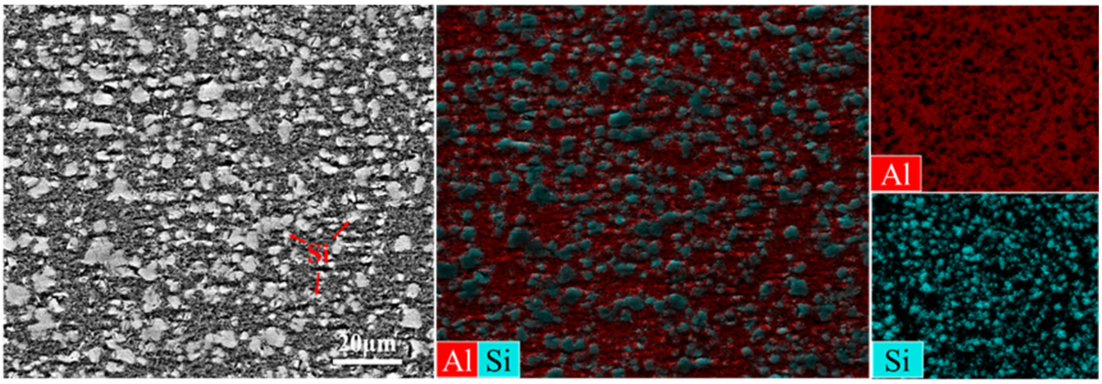

Fig. S1. BSE and EDS characterization of Al-27Si master alloy: eutectic Al-Si matrix and blocky pure Si phase with 3μm average size

Supplementary 2: LA83-0.3Si alloy preparation and processing reference diagram

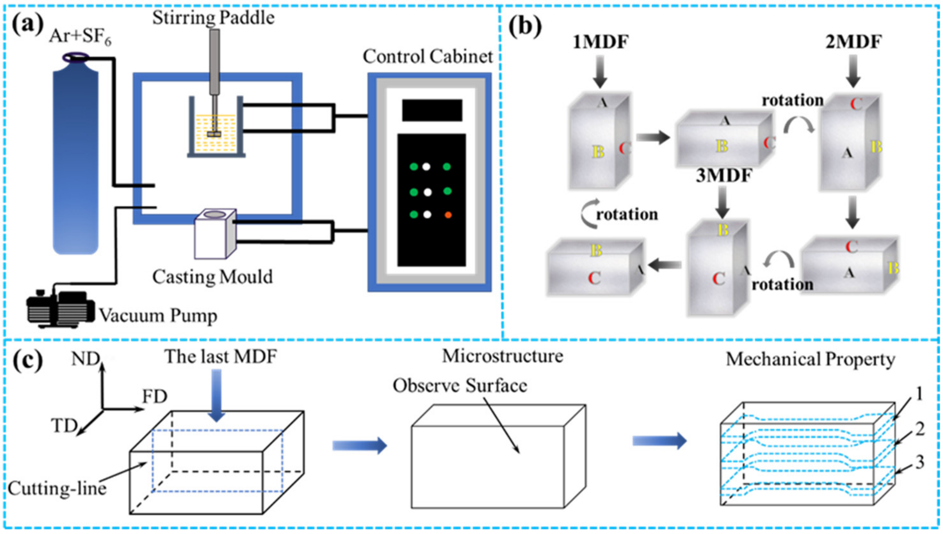

Fig. S2. LA83-0.3Si alloy preparation and processing reference diagram: (a) vacuum resistance heating melting equipment structure diagram, (b) MDF principle diagram, (c) MDF microstructure observation surface and tensile sample selection position map

Supplementary 3: The change of Gibbs free energy of $Li_{22}Si_5$ and Mg_2Si phase formed at 0-600 °C

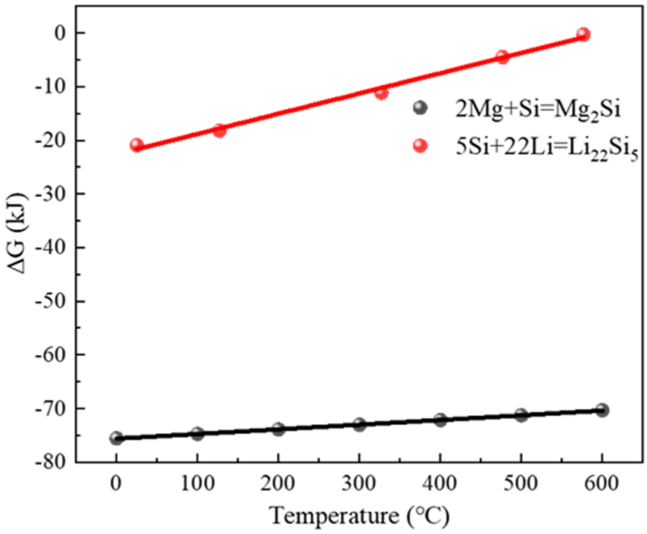

Fig. S3. The change of Gibbs free energy of $Li_{22}Si_5$ and Mg_2Si phase formed at 0-600 °C

Supplementary 4: TEM microstructure of α/β phase boundary in MDF310 alloy

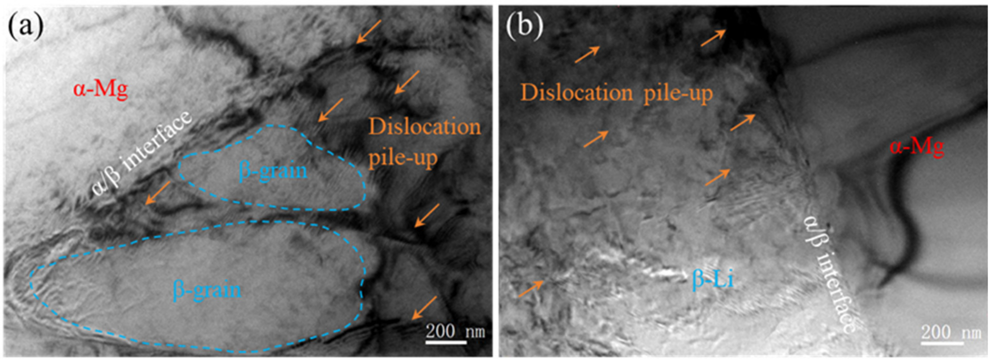

Fig. S4. TEM microstructure of α/β phase boundary in MDF310 alloy
